# Central nervous system biometry in fetuses with and without congenital heart diseases

**DOI:** 10.1007/s00404-022-06484-6

**Published:** 2022-03-17

**Authors:** Aleida Susana Castellanos Gutierrez, Ralf Schmitz, Kerstin Hammer, Janina Braun, Kathrin Oelmeier, Helen Ann Köster, Mareike Möllers, Walter Klockenbusch, Johannes Steinhard, Karol Kubiak

**Affiliations:** 1 Department of Obstetrics and Gynecology, Christophorus-Kliniken GmbH, Coesfeld, Germany; 2 Department of Maternal-Fetal Medicine, Institute Clinic of Gynecology, Obstetrics and Neonatology (ICGON), Hospital Clinic-IDIBAPS, University of Barcelona and Centre for Biomedical Research on Rare Diseases (CIBER-ER), Barcelona, Spain; 3 Department of Obstetrics and Gynecology, University Hospital of Münster, Albert-Schweitzer-Campus 1, 48149 Münster, Germany; 4 Department of Fetal Cardiology, Heart and Diabetes Center North Rhine-Westphalia, Georgstraße 11, 32545 Bad Oeynhausen, Germany; 5 Department of Obstetrics and Gynecology, St. Franziskus-Hospital Münster, Münster, Germany

**Keywords:** Congenital heart disease, Posterior ventricles, Prenatal ultrasound, cavum septi pellucidi

## Abstract

**Objective:**

To compare the fetal brain structures assessed in routine sonographic scans during the second and third trimesters in fetuses with and without congenital heart disease (CHD).

**Methods:**

This is a retrospective cross-sectional single-center study. We measured the head circumference (HC), the transversal diameter of the cerebellum (TCD) and the sizes of the cisterna magna (CM), the cavum septi pellucidi (CSP) and the posterior ventricles (PV) between 20 and 41 weeks of gestation. We compared 160 fetuses with CHD (case group) to 160 fetuses of normal pregnancies (control group). Every patient was matched with a control, considering the gestational age at which the ultrasound was performed. We divided the CHD group into 3 subgroups: retrograde flow in the aortic arch (group 1), right heart anomaly with the antegrade flow in the aortic arch (group 2) and other CHDs with the antegrade flow in the aortic arch (group 3).

**Results:**

The mean width of the PV was larger in fetuses of groups 1 and 3 in comparison to the control group (*P* < 0.001, *P* = 0.022; respectively). We found that the APGAR score at 5 min (*P* < 0.001, *P* < 0.001; respectively) and gestational age at delivery (*P* = 0.006, *P* = 0.001; respectively) were inferior in groups 1 and 3 compared to controls.

**Conclusions:**

Central nervous system biometry is altered in fetuses with CHD. PV is enlarged in CHD fetuses especially with decreased oxygen levels in the aortic arch.

## Introduction

Congenital heart disease (CHD) is the most common congenital malformation diagnosed during pregnancy, accounting for 5–8 births per 1000 live births and constitutes one main risk factor for neonatal mortality and morbidity [1]. Although the survival rates of these children have increased over the last decades, a large proportion of infants with CHD have an adverse neurodevelopmental outcome, including cognitive and motor impairments as well as behavioral and learning problems later in life [2]. Neurological impairments have often been attributed to brain injury from surgical procedures. Recent studies demonstrated signs of abnormal neurological development already present at birth, prior to surgery [3, 4]. These studies demonstrated abnormal results of early neurological examinations and abnormal findings such as periventricular leukomalacia, white matter injury and cerebral atrophy [5].

Under normal conditions, fetal circulation ensures that well-oxygenated and nutrient-rich blood from the placenta is predominantly shifted to the brain. Accordingly, one of the main contributors to abnormal neurodevelopment in fetuses with CHD is probably the presence of altered cerebral perfusion, although this has not yet been proven. Fetuses with CHD display reduced cranial biometry during the third trimester of pregnancy [6]. Different reports describe significantly reduced fetal head biometry in neonates with severe isolated CHD. Smaller head circumference (HC) is mainly reported in neonates with transposition of the great arteries (TGA), tetralogy of Fallot (ToF) and hypoplastic left heart syndrome (HLHS) [7] and is associated with a higher risk for poor neurodevelopmental outcome [8]. Masoller et al. described that abnormal brain development in CHD fetuses could be predicted by midgestational ultrasound features (fetal cranial biometry and Doppler) [4]. The aim of our study is to compare the fetal brain structures assessed in routine sonographic scans during the second and third trimesters in fetuses with and without CHD.

## Patients and methods

In this cross-sectional single-center study, all data were collected and analyzed retrospectively. The study was approved by our institutional ethical review board. Due to the retrospective study design, patient approval or informed consent were not necessary.

All patient data were collected between 2001 and 2018 with the clinical tool Viewpoint®, (General Electric, Wessling, Germany) at the Department of Obstetrics and Gynecology, University Hospital Münster, Germany.

A total of 160 fetuses with CHD and 160 gestational-age-matched fetuses of normal singleton pregnancies (control group) between 20 and 41 weeks of gestation were included. Inclusion criteria for the control group were low-risk pregnancies without maternal or fetal pathologies. Gestational age was determined by the crown-rump length (CRL) in the first trimester of pregnancy. Exclusion criteria for CHD and control group were chromosomal abnormalities, fetal growth restriction, twin pregnancies, unknown outcome of pregnancy, chronic maternal diseases or diseases associated with pregnancy, such as gestational diabetes, macrosomia, gestational hypertension, preeclampsia and eclampsia, or a history of infection during pregnancy.

For the division and classification of the groups, we considered ultrasound images and the distribution of flow in the fetal heart. It is difficult to create a general classification of CHDs to describe brain oxygenation. To the best of our knowledge, there is no publication that has prospectively developed this. All classifications we found were done in the interests of the author. Therefore, we decided to develop a pathophysiological classification. All fetuses with CHD were distributed into three subgroups: Group 1 (a global decrease in oxygen levels in the aortic arch) represents fetuses with a retrograde flow in the aortic arch (50 fetuses), group 2 (physiological oxygenation in the aortic arch) fetuses with a right heart anomaly and antegrade flow in the aortic arch (28 fetuses) and group 3 (different oxygen levels in the aortic arch) all other CHD with the antegrade flow in the aortic arch that could not be categorized into the first two groups (82 fetuses).

Group 1 included fetuses with HLHS (*n* = 48) and aortic stenosis (*n* = 2). Group 2 included fetuses with hypoplastic right heart syndrome (HRHS) (*n* = 23), TGA with aortic stenosis (*n* = 1), ToF with pulmonary stenosis (*n* = 2), right-sided aortic arch with pulmonary stenosis (*n* = 1) and Ebstein's anomaly with pulmonary stenosis (*n* = 1). Group 3 included TGA (*n* = 15), atrioventricular septal defect (AVSD) (*n* = 15), ToF (*n* = 15), atrial septal defect (ASD) (*n* = 12), ventricular septal defect (VSD) (*n* = 6), right-sided aortic arch (*n* = 3), Ebstein's anomaly (*n* = 3), double outlet right ventricle (DORV) (*n* = 3), pulmonary stenosis (n = 2), aortic stenosis (*n* = 2), cardiomegaly (*n* = 2), double aortic arch (*n* = 1), rhabdomyoma (*n* = 1), myocardial hypertrophy (*n* = 1) and diverticulum (*n* = 1).

**Fig. 1:**
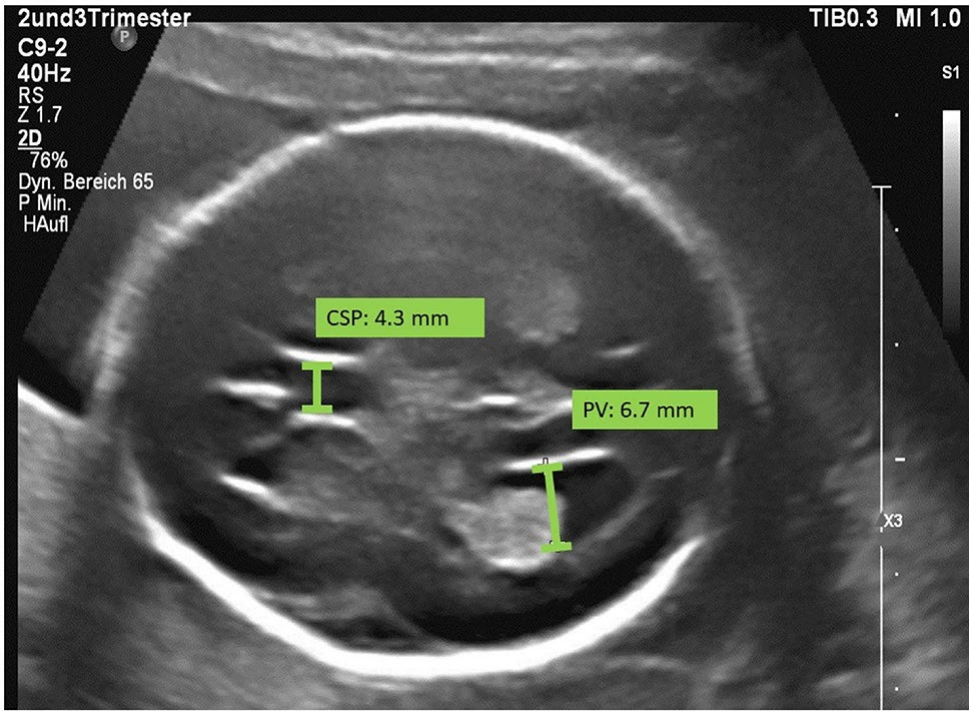
Transventricular plane of a fetus in a normal pregnancy at the 24th week of gestation. The size of the cavum septi pellucidi (CSP: 4.3 mm) and the size of the lateral ventricle (PV: 6.7 mm) were measured

HC, cisterna magna (CM), the transversal diameter of the cerebellum (TCD), and posterior ventricles (PV) were measured according to the ISUOG (International Society of Ultrasound in Obstetrics and Gynecology) guidelines for performing the fetal neurosonogram [9]. The ultrasound images of the axial views of the fetal head were extracted from our database and the values of HC, CM and TCD were used for further analysis. The width of the cavum septi pellucidi (CSP) was quantified in its middle part by placing the calipers on the inner side of the lateral borders as previously described by Abele et al. [10] (Fig. 1). The PV was measured in the same plane. The calipers were placed touching the inner edge of the ventricle wall at its widest part and aligned perpendicular to the long axis of the ventricle [9]. The measurements of fetal biometry parameters were performed by physicians specialized in maternal–fetal medicine; in a few cases, the size of PV and CSP were not been saved at the consultation period, therefore the measurements were done by an observer (A.S.C.) retrospectively from stored images. The fetal outcome parameters (gestational age at delivery, birth weight, APGAR score after 5 min, pH of umbilical artery, HC at birth) were recorded for all cases (Table 1).

**Table 1:** Characteristics of the study population and comparison of the parameters in cases of CHD to the control group

Parameter	Control group (*n* = 160)	Group 1 (*n* = 50)	Group 2 (*n* = 28)	Group 3 (*n* = 82)	P1	P2	P3
BPD (mm)	78.2 (63.3, 87.7)	87.6 (62.9, 93.1)	78.9 (65.9, 88.5)	71.2 (57.9, 82.4)	0.570	0.948	0.611
FOD (mm)	98.9 (79.0, 108.2)	107.6 (79.6, 112.0)	96.9 (84.7, 105.2)	92.3 (73.7, 105.0)	0.226	0.640	0.715
HC (mm)	280.5 (223.5, 311.7)	308.0 (221.5, 320.0)	279.5 (237.2, 312.7)	251.0 (207.5, 295.2)	0.738	0.974	0.623
CM (mm)	6.6 (5.8, 7.4)	6.6 (5.8, 8.0)	6.8 (5.6, 7.6)	6.3 (5.6, 7.2)	0.430	0.574	0.638
TCD (mm)	36.1 (27.1, 44.0)	39.7 (27.2, 45.5)	36.6 (28.8, 42.5)	32.7 (24.5, 39.9)	0.417	0.768	0.583
PV (mm)	4.7 (3.7, 6.1)	5.6 (4.4, 6.9)*	5.3 (3.6, 6.0)	5.5 (4.3, 6.8)	0.007	0.799	0.068
CSP (mm)	4.9 (4.0, 5.6)	5.2 (3.8, 5.6)	4.6 (3.9, 5.3)	4.6 (3.6, 5.2)	0.563	0.570	0.428
5-min APGAR	10 (9, 10)	9 (8, 9)*	9 (8, 10)	9 (8, 10)*	< 0.001	0.063	< 0.001
GA at examination (weeks)	29.6 (24.4, 34.8)	34.5 (25.2, 36.4)	29.7 (25.3, 33.5)	28.0 (23.5, 32.1)	1.000	0.964	0.979
GA at delivery (weeks)	39, 7 (38.2, 40.5)	38, 7(37.4, 40.1)*	39, 2 (38.1, 40.0)	38, 5 (37.7, 39.5)*	0.006	0.688	0.001
Weight at examination	1541 (680, 2440)	2273 (800, 2893)	1424 (856, 2245)	1087 (577, 1871)	0.736	0.831	0.671
Birth weight(g)	3272 (2980, 3610)	3270 (2767, 3605)	3240 (2783, 3700)	3150 (2855, 3512)	0.684	0.654	0.077
Birth HC (cm)	34.5 (33.5, 35.5)	34.0 (32.0, 35.5)	33.0 (32.0, 34.0) *	34.0 (32.3, 35.0)*	0.159	0.025	0.004
pH umbilical artery	7.29 (7.23, 7.34)	7.28 (7.21, 7.33)	7.27 (7.22, 7.31)	7.30 (7.24, 7.33)	0.817	0.081	0.677

Data presented as median (interquartile range)

*P*-values from Mann–Whitney-*U*-Test; *BPD* biparietal diameter, *FOD* fronto-occipital diameter, *HC* head circumference, *CM* cisterna magna, *TCD* transversal cerebellar diameter, *PV* lateral ventricle measurements, *CSP* cavum septipellucidi, *APGAR* Appearance, Pulse, Grimace, Activity and Respiration, *GA* gestational age

*Group 1* retrograde flow in the aortic arch, *Group 2* right heart anomaly with antegrade flow in the aortic arch, *Group 3* other CHDs with antegrade flow in the aortic arch

*P1* Control group vs. group 1, *P2* Control group vs. group 2, *P3* control group vs. group 3. Note that *P*-values were computed using only those controls that were matched to the cases of the CHD group under consideration

^*^P-values < 0.05 were considered indicative of statistical noticeability

### Statistical analysis

All data were analyzed with SPSS software (IBM Corporation, New York, NY, USA, version 25). Descriptive statistics were used to characterize the study population. Normally and not normally distributed parameters are shown as median and interquartile range. Differences in general characteristics between the case and control groups were assessed using Mann–Whitney-*U*-Test. No adjustment for multiple comparisons was performed. All analyses have to be considered exploratory, and *P*-values were merely considered indicative of statistical noticeability in the case of *P* ≤ 0.05. Multivariate linear regression was used to include gestational age as an additional variable in comparisons between cases and controls.

## Results

The characteristics of the study population and the comparison of the parameters in cases of CHD to the control group are visualized in Table 1.

We found a statistically noticeable increased width of PV in fetuses of group 1 compared to the control group (5.6 mm (4.4, 6.9) vs. 4.7 mm (3.7, 6.1), *P* < 0.007). Further, it could be shown that the PV in group 2 (5.3 mm (3.6, 6.0), *P* = 0.799) and 3 is also enlarged (5.5 mm (4.3, 6.8), *P* = 0.068).

The examined biometric parameters BPD, HC, CM, TCD and FOD did not show any statistical differences. In group 3 we observed a trend to lower measurements for all biometric parameters.

For fetal outcomes at birth, there are differences comparing the control group against group 1 and 3 regarding the APGAR score after 5 min (*P* < 0.001, *P* < 0.001; respectively).

The gestational age at delivery was lower in group 1 and 3 in comparison to the control group (*P* = 0.006, *P* = 0.001; respectively).

We observed a decreased HC at birth in all groups with CHD. In group 2 and 3 our findings compared to the control group were statistically noticeable (*P* = 0.025, *P* = 0.004; respectively).

Weight at birth for the fetuses in all groups showed no statistically noticeable differences, nevertheless group 3 showed lower weight at birth compared to the control group (3150 g vs. 3272 g; *P* = 0.077).

The CSP in group 1, 2 and 3 were comparable to the control group (*P* = 0.563, *P* = 0.570, *P* = 0.428; respectively).

The results of the multivariable linear regression of PV on gestational age and CHD/control group are shown in Table 2 and reaffirm the results of the univariate analysis. Additionally, we found a statistically noticeable increased PV in group 3.

**Table 2:** Results of the multivariable linear regression of PV and CHD/control group

Parameter	Regression coefficient	*P*	95% confidence interval	
			Lower	Upper
Gestational age (weeks)	− 0.106	< 0.001	− 0.137	− 0.074
Group 1 vs control	0.936	< 0.001	0.426	1.447
Group 2 vs control	0.276	0.391	− 0.355	0.906
Group 3 vs control	0.493	0.022	0.073	0.914

*PV* lateral ventricle measurements, *CHD* congenital heart disease, *Group 1* retrograde flow in the aortic arch, *Group 2* right heart anomaly with antegrade flow in the aortic arch, *Group 3* other CHDs with antegrade flow in the aortic arch

## Discussion

In our study fetuses with CHD and especially those presenting a retrograde flow in the aortic arch showed larger PV-size in comparison to healthy fetuses. Those fetuses had been diagnosed predominantly with HLHS. The other neurosonography biometry parameters were comparable for all groups.

Regarding neonatal parameters, we found that the APGAR score at 5 min and gestational age at delivery were decreased in CHD fetuses with retrograde flow in the aortic arch and other CHDs with antegrade flow in the aortic arch. HC at birth was reduced especially in right heart anomaly fetuses with antegrade flow in the aortic arch and other CHDs with antegrade flow in the aortic arch.

PV measurement is important to assess proper brain development, as its alteration might lead to the diagnosis of ventriculomegaly (VM). VM is defined as ventricles larger than 10 mm, and can occur bilaterally or unilaterally [11, 12]. VM has been related to other brain anomalies; however, it can also be isolated without a pathological significance.

VM can result from different processes. One is the abnormal turnover of cerebrospinal fluid (CSF), as an imbalance between production and absorption. On the other hand, VM can be due to disorders in neurodevelopment, for instance structural and metabolic disorders [13].

In our study, altered PV measurements were below the definition of VM. In previous studies, we described a slight dilatation of PV associated with diabetes mellitus in pregnancy [14]. Minova et al. considered slightly abnormal PV clinically relevant because they found that slight PV enlargement in newborn infants was associated with respiratory disturbances, feeding hypoxemia and diabetes mellitus [15]. Nevertheless, to our knowledge these findings are sporadic and not well described in the literature.

We hypothesize the dilatation of PV seen in the fetuses of our patients could be associated with chronic hypoxia and reduction of brain metabolism. The PV sizes are statistically noticeable larger for group 1 and 3, However, due to the minor expansion of the lateral ventricle, its clinical significance is questionable.

Several studies have shown an association between CHD and smaller HC. Microcephaly is defined by the measurement of occipital-frontal circumference (HC) more than 2 standard deviations (SDs) below the mean for age and sex [16]. Severe microcephaly is described as head circumference more than 3 SDs below the mean for age and sex [17]. Barbu et al. observed that microcephaly of newborn infants was associated with significant hemodynamic changes in the fetal cerebral circulation [18]. The pathophysiology is related to reduced oxygen content resulting from altered cardiac mixing of oxygenated and deoxygenated blood as a result of cardiac abnormalities. As a response, a compensatory vasodilation is induced, which increases cerebral blood flow and oxygen consumption. A final consequence of reduced cerebral oxygenation could be an impaired brain development [19]. A systematic review published in 2017 showed a correlation between congenital heart defects and reduced prenatal brain growth [20]. Another study found that the presence of HLHS could also have a negative effect on brain development and lead to a higher rate of microcephaly at birth [18]. A retrospective study by Graupner et al., including 248 CHD fetuses, showed no differences between fetuses with isolated CHD and HC growth. However, a subgroup analysis of fetuses with retrograde aortic arch flow and therefore with low oxygenated brain blood flow, showed a decreased growth of HC in the 3rd trimester [21]. In our CHD collective the HC was not yet significantly smaller prenatally. Postnatal it was statistically noticeable reduced in groups 2 and 3, but this can be explained by the difference in mean gestational age at birth.

The CSP has been measured and correlated to different perinatal pathologies [22]. In our previous study, we found that the width of the CSP has a direct correlation with diabetes [14]. Nevertheless, in our study we found comparable results for CSP in all groups after matching with gestational age. In contrast, two studies described enlarged CSP associated with isolated congenital heart disease, for example in fetuses with HLHS and dextro-transposition of the great arteries [23, 24].

The examined biometric parameters BPD, HC, CM, TCD and FOD did not show any statistically differences but the measurements of group 3 are noticeably lower. These could also be found in the lower noticeable birth weight in group 3. The reason for these differences is unclear, but we assume a general hypoxic situation similar to early mild pre-eclampsia with small for gestational age fetuses.

A limitation of our study is the retrospective design and the heterogeneous types and manifestations of heart defects. It is necessary to perform a consistent allocation of subgroups to compare the results of different studies. Therefore, it is relevant that we all “speak the same language”. Furthermore, a structured follow-up protocol of postnatal neurodevelopment is of clinical importance. Moreover, prospective studies could be done to assess the effect of neurological development supported by specific medical and health care for CHD patients.

Despite the limitations, the strength of the study is that we collected data from a large number of cases with CHDs. Therefore, we can enhance the validity of our results.

However, we collected data from a large number of cases with CHDs. In the future prospective studies must be done to assess neurological development of patients with a history of CHD during pregnancy.

## Conclusions

Central nervous system biometry is altered in fetuses with CHD. PV is enlarged in CHD fetuses especially with decreased oxygen levels in the aortic arch. A possible pathophysiology, which may explain the altered brain development or cranial biometrics in CHD fetuses, remains unclear. Further research on those interactions should be carried out.
